# Host susceptibility to severe influenza A virus infection

**DOI:** 10.1186/s13054-019-2566-7

**Published:** 2019-09-05

**Authors:** Sara Clohisey, John Kenneth Baillie

**Affiliations:** 1 Division of Genetics and Genomics, Roslin Institute, University of Edinburgh, Easter Bush, Edinburgh, EH25 9RG UK; 2 Intensive Care Unit, Royal Infirmary of Edinburgh, 54 Little France Drive, Edinburgh, EH16 5SA UK

**Keywords:** Influenza, ARDS, Susceptibility, Genetics

## Abstract

Most people exposed to a new flu virus do not notice any symptoms. A small minority develops critical illness. Some of this extremely broad variation in susceptibility is explained by the size of the initial inoculum or the influenza exposure history of the individual; some is explained by generic host factors, such as frailty, that decrease resilience following any systemic insult. Some demographic factors (pregnancy, obesity, and advanced age) appear to confer a more specific susceptibility to severe illness following infection with influenza viruses. As with other infectious diseases, a substantial component of susceptibility is determined by host genetics. Several genetic susceptibility variants have now been reported with varying levels of evidence. Susceptible hosts may have impaired intracellular controls of viral replication (e.g. IFITM3, TMPRS22 variants), defective interferon responses (e.g. GLDC, IRF7/9 variants), or defects in cell-mediated immunity with increased baseline levels of systemic inflammation (obesity, pregnancy, advanced age). These mechanisms may explain the prolonged viral replication reported in critically ill patients with influenza: patients with life-threatening disease are, by definition, abnormal hosts. Understanding these molecular mechanisms of susceptibility may in the future enable the design of host-directed therapies to promote resilience.

## Introduction

The normal response to infection with influenza A virus (IAV) is to remain asymptomatic. During the 2009/2010 pandemic, serosurveillance studies revealed that a majority of volunteers who tested positive for antibodies to the new H1N1pdm09 virus did not report any symptoms [1]. The majority of people newly exposed to one of the most dangerous viruses to circulate in human populations in recent history, which in the same population created an overwhelming burden of critical illness [2], did not notice any symptoms.

Wide variation in susceptibility is a general feature of human and animal populations exposed to any pathogen [3]. Explaining the mechanisms of susceptibility may enable effective targeting of vaccine therapies, may reveal new therapeutic approaches [4, 5], and, in theory, may contribute to future clinical risk prediction models.

## Variation attributable to the virus

## Initial exposure

As with any infectious disease in a given host, the site of infection, the scale of the initial exposure, and the virulence, degree of pathogenicity, of the pathogen determine the nature of the disease in IAV infection. Although the alimentary tract is a common site of infection in other species (for example the natural hosts, water fowl [6]), initial infection in humans is through the respiratory tract. The number of viable IAV virions transmitted has a direct effect on the probability of symptoms, both in animal models [7] and human challenge studies [8]. This may explain a proportion of the variation in individual responses to the virus.

## Virulence

The virulence of the virus itself varies greatly. Perhaps fortunately, there is a general trend for the most virulent IAV strains to be less transmissible; that is, those that cause the most severe disease are less likely to be passed on to others. While highly transmissible IAV strains, such as H1N1pdm09, replicate well in the upper respiratory tract, viruses associated with higher rates of severe disease, such as H5N1 and H7N9 avian IAV, exhibit tropism for the lower respiratory tract [9, 10].

Within a given strain, not all IAV viruses are the same. In fact, it is statistically unlikely that *any* two IAV virus particles will have exactly the same genome sequence. Small changes, such as a single amino acid change in the hemagglutinin protein, can significantly alter the tropism of the virus for example, increasing the likelihood of spread to the lower respiratory tract and establishing a more severe infection [11].

IAV viruses change rapidly by two mechanisms: shift and drift. Shift is the exchange of viral segments between strains, occasionally resulting in a new IAV subtype to which a large proportion of the population does not have existing immunity. This shuffling of the viral genes contributes to the sudden and dramatic change in virulence that may occur from season to season, and to zoonoses, as IAV jumps from its natural avian host to mammalian pig and human hosts.

Drift refers the accumulation of small mutations in the viral genome that occur on a continuum. Because of the short genome (around 13,500 bases of RNA are carried by a functional virion particle) and a very high error rate when this genome is replicated [12, 13], viral quasispecies arise, leading to a heterogenous swarm of virions [14]. This variation enables IAV to evolve extremely rapidly where a selective pressure exists. For example, it is likely that IAV can evolve de novo resistance to antivirals during treatment of a single patient [15–17].

Studies of viral whole genome sequence during outbreaks have failed to identify consistent viral factors associated with severe disease [18]. It is therefore likely that viral factors do not explain the vast spectrum of variation observed in the disease.

## Variation attributable to the host

## Previous exposure to IAV

Due to the remarkable memory of the adaptive and innate immune systems, previous exposure to IAV has a strong effect on future susceptibility. Adaptive immune memory is highly strain-specific and provides targeted antibody-mediated defence against IAV [19].

The first IAV strain to which a child is exposed has a profound effect on subsequent immunity—a concept known as *original antigenic sin* [20]. The host immune system is extensively programmed by this first IAV exposure, such that the susceptibility of whole populations of adults can be predicted using the patterns of circulating IAV in each patient’s year of birth [21]. This has been proposed as one reason why the burden of mortality for the 2009/2010 outbreak was shifted towards patients younger than 65 years of age [22]—patients over 65 years old are more likely to have been exposed at a young age to an IAV strain similar to the H1N1pdm09 strain, and were hence protected.

Interestingly, the lifelong immunity provided by this first IAV exposure has broad protective effects against different IAV strains [21]. Cell-mediated immunity may play an important role in this protection. An IAV challenge study in healthy volunteers found that pre-existing CD4(+) T cell responses to IAV nucleoprotein and matrix proteins were present prior to infection [23]. The magnitude of this CD4(+) T cell response when challenged correlated with reduced symptoms and reduced virus shedding.

## Host demographics

**Fig. 1:**
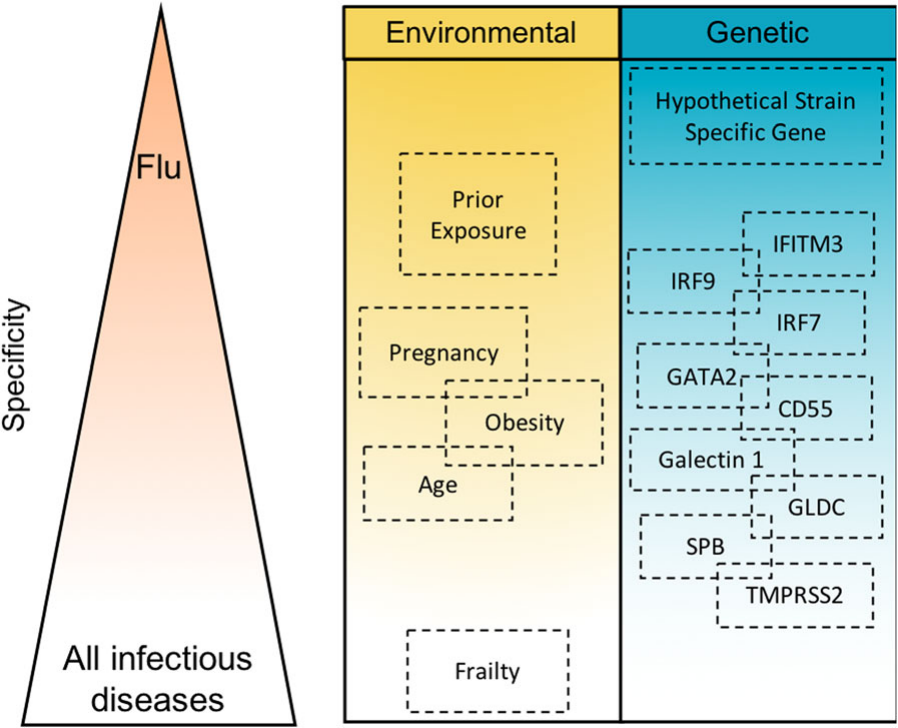
Conceptual visualisation of variation in specificity of host susceptibility factors. Factors predicted to confer more specific susceptibility to influenza are placed higher in the diagram

Regardless of prior exposure, the most reliably quantified risk factors for life-threatening seasonal and pandemic IAV are advanced age (> 65 years), obesity, immunosuppression, cardiovascular disease, and neuromuscular disease [24]. A number of well-recognised host factors—best summarised by the broadly understood but poorly defined term, “physiological reserve”—increase the chance of organ failure and death following any severe injury or infection. These factors are extensively discussed elsewhere in the critical care literature; here, we focus on host factors that are thought to confer some element of specific susceptibility to IAV (Fig. 1).

### Pregnancy

Studies dating back to the 1918–1919 pandemic have suggested that pregnancy, particularly in the third trimester, increases the risk of death from IAV [25]. Additionally, pregnant women have a higher rate of hospitalisation with seasonal IAV [26]. However, in the largest systematic review of clinical risk factors for IAV, pregnancy was not independently associated with severe disease from either seasonal or pandemic IAV [24].

The immunological changes that occur in pregnancy are theoretically compatible with increased severity of IAV: in particular, an increase in innate immune activation and a decrease in the number and activity of cells associated with cytotoxic immunity—in which infected cells are killed to limit the dissemination of the virus [27]. These changes may lead to an increased propensity to develop ARDS [28] and a decreased ability to eliminate IAV-infected cells, which is a core component of anti-IAV immunity.

Some indices of severity used in epidemiological studies are themselves directly affected by pregnancy. The cardiovascular adaptations to pregnancy, combined with an increased metabolic rate, a decrease in functional residual capacity, and increased basal ventilation to perfusion mismatch, are expected to worsen hypoxaemic respiratory failure following any insult. In parallel, admission to hospital or critical care may be in part biased by elevated concern for a pregnant patient, and by the perception of high risk of severe IAV [29].

### Obesity

Obesity was identified as a risk factor for IAV infection over a decade ago and confirmed during the swine flu pandemic [30, 31] when it was associated with an increased risk of death [32]. Although comorbidities associated with obesity—specifically diabetes mellitus and cardiovascular disease—compromise pulmonary host defence and increase the chance of death following any severe systemic injury [33], an independent association between obesity and severe IAV is robust and replicated [24].

In parallel with the immune changes associated with pregnancy, obese patients are more likely to have impaired cell-mediated immunity and excessive chronic activation of the innate immune system [34]. This is reflected in a study which demonstrated that among vaccinated adults, those who are obese are more likely to suffer severe consequences of IAV [35]. Furthermore, it has been shown that obese adults have an impaired antibody response to IAV vaccination [36], and impaired CD4(+) and CD8(+) T cell responses IAV in vitro [37]. Obese patients have a prolonged period of viral replication and shedding, even in the absence of clinical disease [33].

### Age

Extremes of age are well-recognised risk factors for severe disease. Children under the age of 5 years, and particularly those under 2 years, have consistently been found to be at high risk for severe disease and serious complications following IAV infection [38–40]. Functional immaturity of the immune system, together with a failure to recognise IAV-related antigens, may largely explain this effect.

In industrialised countries, the group at highest risk of death from seasonal IAV is those over 65 years of age [22, 41, 42]. Senescence affects antiviral immunity in complex ways; it is difficult in clinical epidemiological studies to distinguish the effect of these immune changes from the effects of frailty and antigenic exposure. Baseline markers of systemic inflammation are elevated [43] and circulating T cell counts are reduced. Naive T cells, a key component of cell-mediated adaptive immunity, are lost from the circulation due to the process of thymic involution, which begins very early in life [44]. In mouse models of IAV infection, aged mice exhibit slower antiviral and adaptive immune responses, and more severe disease [45].

Expansion of clonal T cell populations, driven by cytomegalovirus (CMV), occurs in older adults and may impair T cell responses to new pathogens [46]. In contrast, in the young, a multi-omic systems analysis demonstrated that CMV infection is associated with an enhanced T cell mediated response to IAV vaccination [47]. Integrating systems studies of host response to IAV infection with markers for genetic susceptibility (see below) may in the future reveal new biological pathways and patterns of disease [48].

As with pregnancy and obesity, ageing is associated with both an increase in the basal activation of the innate immune system (sometimes referred to as “inflammaging”) and a decrease in cell-mediated immunity. This combination of mechanisms may explain the particularly strong effects on susceptibility.

## Host genetics

Susceptibility to death from any infection is strongly inherited by children from their parents [49]. In IAV, numerous genetic studies in humans and animal models have revealed specific genes associated with susceptibility, which are extensively reviewed elsewhere [50–52]. In addition to the specific genetic variants discussed below, there is direct evidence, from a study of death records in Utah, that susceptibility to IAV is heritable at a population level [53].

### Inborn errors of immunity

Much of what is known about human genes associated with IAV susceptibility has been discovered from loss-of-function mutations in the immune system, which lead to loss of the gene product or a substantial reduction in gene function. These often lead to severe defects that are likely to present in childhood. Such variants can reveal key components of the immune response to a specific infection. In considering the biological lessons from such discoveries, it is important to consider that, in most people, these components of the immune system function perfectly well and may not be suitable targets for therapy. Secondly, there is little that can be inferred from the *absence* of any particular gene, or immune process, from the list of loss-of-function defects associated with susceptibility to IAV.

The conditions that must be met for such a gene to be discovered are not restricted to disease susceptibility. Many variants that confer susceptibility to IAV have broader pleiotropic effects that may be terminal in utero or in early life, or may lead to susceptibility to other infections or autoimmune conditions that obscure the clinical picture. Alternatively, some variants may lead to strain-specific susceptibility and will only be detected following exposure to the right virus.

The full range of genetic defects associated with susceptibility to IAV in animal models is reviewed elsewhere [54, 55]. So far, three known human genes, all transcription factors active primarily in myeloid cells, have been found to have loss-of-function variants that increase susceptibility to IAV. Since transcription factors function as master regulators of large numbers of genes, functional deficiencies are expected to have broad, non-specific effects.

#### IRF7

In 2015, Ciancanelli et al. identified a patient with a mutation in the transcription factor Interferon regulatory factor 7 (IRF7) that led to severe infection and ARDS when she was 2.5 years old [56]. IRF7 is a transcription factor and a key regulator of the type I interferon response. This was the first published example of a single-gene inborn error of immunity that was specific to IAV. Both parents were heterozygous for *different* loss-of-function alleles, but each had sufficient functional IRF7 activity allowing them to avoid severe IAV. The patient inherited these two different loss-of-function alleles (compound heterozygosity) leading to complete loss of functional IRF7. Leukocytes and plasmacytoid dendritic cells from this patient produced very little interferon type I (*α*/*β*) and III (*γ*) in vitro indicating that expression and production of these interferons in these cell types is specifically IRF7-dependent in IAV infection in humans.

#### IRF9

Whole exome sequencing of 20 children identified a variant in the gene encoding interferon regulatory factor 9 (IRF9) in a 2-year-old child who had previously suffered from bronchitis and biliary perforation [57]. The child inherited a mutation on both alleles from consanguineous parents leading to a single change in the DNA sequence (single nucleotide polymorphism, SNP) in the IRF9 gene. This SNP occurs at an essential site leading to aberrant processing of the gene transcript and thus the expression of a truncated, functionally defective, protein product.

In this case, IRF9 was only partially defective. Activation of interferon-stimulated gene 3 (ISG 3) was impaired in response to IAV infection or interferon *α* stimulation, but other IRF9-dependent pathways remained intact. The consequence of this appears to be a global reduction in type I interferon responses, a key mechanism of early mucosal resistance to infection, in all cell types. Unrestricted viral replication was observed in cells from the patient and was also shown for parainfluenza virus and respiratory syncytial virus.

#### GATA2

GATA2 is a zinc finger transcription factor, part of the GATA family, so named because they bind a G-A-T-A pattern (also called a motif) in DNA sequence. Transcription factor binding at sites bearing this motif alters the probability that a given gene will be transcribed, and ultimately controls the amount of the encoded protein that is made. GATA2 deficiency results in primary immune cell deficiency and affects a wide range of cell types. Decreased circulating counts of B lymphocytes, NK cells, monocytes and plasmacytoid dendritic cells have been observed, along with reduced T cell thymic output. In 2018, Sologuren et al. published a case study of a father and son who contracted and subsequently died from severe IAV [58]. Both patients were heterozygous for a novel mutation in GATA2 that led to a dysfunctional protein.

Despite the known effects of GATA2 deficiency on primary immune development, the first, older patient had suffered few health problems prior to his 30th year, after which frequent respiratory illnesses and a single incidence of viral pneumonia is reported prior to his severe illness. The second patient had been hospitalised with pneumonia at 16 without recurrence until hospitalisation with severe IAV at 31. The authors attribute protection from viral and bacterial infection observed in the lifetime of these patients to long-living memory T and B cells.

### Population genetic studies

Genetic variants with less drastic effects on susceptibility can be detected by comparing flu-susceptible populations with control populations (Table 1). These studies generally look for candidate genes or take a genome-wide approach.

**Table 1:** Genes and associated single nucleotide polymorphisms (SNPs) related to influenza A susceptibility in humans

Gene	Gene name	Function	SNP	Reference
Entry factors/cell membrane				
IFITM3	Interferon-induced transmembrane protein 3	Antiviral	rs12252-C	[64–66]
			rs34481144-A	[67]
CD55	Complement decay-accelerating factor precursor 55	Inhibition of complement activation	rs2564978 T/T	[71, 72]
TMPRSS2	Transmembrane protease, serine 2	Serine protease	rs2070788 GG	[73–76]
GLDC	Glycine decarboxylase	Component of the glycine cleavage system	rs1755609-G	[80]
LGALS1	Galectin-1	Cell-cell interactions	rs4820294	[82]
			rs2899292	[82]
			rs4820294	[82]
ST3GAL1 (*)	ST3 beta-galactoside alpha-2,3-sialyltransferase 1	Transfer of sialic acids to galactose-containing substrates	rs113350588	[90]
			rs1048479	[90]
TNFA (*)	Tumour necrosis factor alpha	Inflammation and immune signalling	rs361525-A	[91]
TLR3 (*)	Toll-like Receptor 3	Pathogen recognition	rs5743313-CT	[92]
			rs5743313-CC	[72]
Surfactant proteins				
SP-A2 (*)	Pulmonary-surfactant associated protein A2	Pathogen binding and immune signalling	rs1965708-C	[89]
			rs1059046-A	[89]
SP-B	Pulmonary-surfactant associated protein B	Pathogen binding and immune signalling	rs1130866	[77]
Interleukins				
IL1A (*)	Interleukin 1 alpha	Inflammation and immune signalling	rs17561-T	[84]
IL1B (*)	Interleukin 1 beta	Inflammation and immune signalling	rs1143627-C	[84]
			rs16944-AG	[85]
			rs3136558-TC	[85]
IL28B	Interleukin 28 b, IFN-λ 3	Immunomodulation	rs8099917-TT	[86]
IL17 (*)	Interleukin 17	Inflammation and immune signalling	rs2275913 (GG and AG)	[87]
IL6 (*)	Interleukin 6	Inflammation and immune signalling	rs1818879-(GA and GG)	[85, 88]

Gene: gene symbol. Gene name: gene name and alternate name. Function: summary function of gene product. SNP: SNPs associated with host susceptibility to influenza A associated with gene. (*) represents genes not addressed in the text

Candidate gene association studies have a long but troubled history in human genetics. Genes are selected because of some underlying hypothesis; single variants within these genes are then chosen because they are believed to have an effect on the expression or function of the gene. Genotype frequencies (that is, the proportion of a population who have a given variant) at these genomic positions are then compared between a case and control group. This has the advantage of economy, since only one or two variants need to be genotyped for each participant, and has the superficial appearance of statistical efficiency, since fewer comparisons are made.

The fundamental limitation is that, in a human genome composed of 3 × 10^9^ bases, of which 4 − 5 × 10^6^ are different between any random pair of people [59], the probability of choosing the right base is very low. In the event that a given variant meets a nominal level of significance, the evidence for an association is easily misinterpreted. Looking backwards from a single small *p* value, it is common to focus on the fact that probability of seeing such an association by chance alone is very low. What is easy to forget is that the probability of such an association existing is also very low.

An understanding of this methodology is important for the interpretation of such studies. Many of the positive studies reflect more the biases of well-informed investigators in the choice of target genes. The additional value of an unreplicated genetic association on this background is often small.

Nonetheless, candidate gene approaches in various forms detected numerous real and informative associations with disease before the advent of genome-wide genotyping technology [60]. We focus here on larger studies, those that have been replicated, and studies with particular relevance to the pathogenesis of severe IAV.

Genome-wide approaches seek to eliminate the aforementioned bias. In the most widely used design, the genome-wide association study (GWAS), hundreds of thousands of common variants are genotyped in each patient. This is expensive and requires correction for multiple comparisons. A widely used convention is to correct for 1 × 10^6^ independent comparisons in each study, requiring a *p* value <5 × 10^−8^ for significance. Large numbers of patients are needed to detect associations at this level above the background noise of variation in human populations. However, genome-wide approaches use no preconceptions about the pathogenesis of disease. Hence, such methods have the potential to teach us something that we did not already know. Because of the stringent threshold for statistical significance, and the burden of multiple testing, statistical power to detect small effects is usually lacking unless many tens of thousands of patients are included. For this reason, the expected outcome is false-negatives. Hence, we would caution against drawing any conclusion from the *absence* of significant associations within a given gene.

Genome-wide in vitro knockdown screens can also be used to limit bias and enable genome-wide discovery. In this approach, although a candidate gene is often selected from cell culture results and tested for genetic associations in patients, there is an important difference from single-gene candidate studies: the pool of genes from which the candidate is chosen comprises the entire protein-coding part of the genome.

#### Intracellular antiviral immunity IFITM3

A role for interferon-induced transmembrane protein 3 (IFITM3) in IAV replication was discovered in an in vitro genome-wide knockdown screen in cultured cells [61]. The protein product of this gene restricts IAV entry by blocking the fusion of host and viral membranes [62] and acts as a restriction factor in viral infections, along with family members IFITM1 and IFITM2 [61]. IFITM proteins were also shown to inhibit the early replication of other virus types, for example the West Nile Virus [63].

Based on this genome-wide knockdown screen, a candidate gene sequencing approach was conducted to test for an association with severe illness. The 2009/2010 pandemic provided a colossal natural experiment—a large proportion of the population were exposed to a new pathogen, but only a small minority developed life-threatening illness requiring critical care. Focusing on these previously healthy adults with life-threatening IAV (in the GenISIS and MOSAIC studies) may have increased the effect size seen [64].

Genotypes at every variant within the IFITM3 gene were compared with population controls, identifying a single variant (rs12252-C) associated with severe IAV. This variant is rare in the European cohorts in which it was discovered, but is frequent in the Han Chinese cohorts hospitalised with severe H1N1pdm09 infection [65]. The association has been replicated in independent studies in different populations [66].

A second SNP associated has been shown in population-level studies to regulate IFITM3 expression. rs34481144-A encourages the transcription factor CTCF to bind to the regulatory region of IFITM3 and repress gene expression in response to IAV infection [67]. This SNP can also disrupt the methylation pattern (a key modification of DNA that usually silences genes) in the regulatory region leading to cell type-specific effects. IFITM3 expression in memory CD8(+) T cells in response to viral infection has been found to protect and encourage survival of these cells allowing for the establishment of adaptive immunity. Loss of methylation at this site prevents CTCF from binding to the DNA and inducing expression of IFITM3 in response to the pathogen, thus reducing cell survival. This is estimated to lead to a 2.6-fold increased risk of a severe outcome upon IAV virus infection. IFITM3 has also been recently shown to have a protective effect on the heart during severe IAV infection. Myocarditis has been associated with IAV infection since the 1918 pandemic [68], and IAV has been shown to lead to a sixfold increased risk of myocardial infarction in the 7 days post infection [69]. So far, IFITM3 is the only gene for which SNPs have been identified and independently confirmed in vivo and in vitro to restrict IAV replication [70]. However, this gene is not specific to IAV replication and the full extent of the antiviral actions remains to be discovered.

#### Immune-myeloid/T cell CD55 decay accelerating factor

Unbound complement is rapidly inactivated in plasma. Where this process is defective, uncontrolled complement activation can damage host cells.

CD55 prevents the formation and accelerates the decay of C3 and C5 convertases. These proteases are part of the complement system and have roles in opsonisation and the release of inflammatory molecules. CD55 polymorphisms were associated with severe H1N1pdm09 infection (defined as requiring supplementary oxygen, admission to intensive care or death) [71]. This study found carriers of the rs2564978-T/T polymorphism had significantly lower levels of surface CD55 on their circulating monocytes compared to the more common C allele. Further work identified a deletion in a nearby regulatory region as the element responsible for the specific effect on both protein and mRNA levels of CD55 in monocytes. A more recent study of Han Chinese individuals that looked at several genes confirmed an association between CD55 rs2564978 T/T and death from severe IAV infection [72].

The cumulative effects of multiple SNPs (IFITM3, CD55, and the immune cell receptors TLR3 and TLR4) on IAV susceptibility have been examined in a targeted study [72]. This independently confirmed the association of the CD55 rs2564978 polymorphism with severity, and the IFITM3 rs12252-C and TLR3 rs5743313-CC genotypes were both over represented in fatal cases.

#### TMPRS22

In a small-scale pilot study, genome-wide genotypes of 42 patients with severe IAV were compared to 42 controls with mild IAV. The rs2070788-G allele of TMPRS22 was significantly overrepresented in severe compared with mild cases of H1N1pdm09, with a > 2-fold higher risk of severe infection. There was higher TMPRS22 expression in human lung tissues with the high-risk GG genotype [73]. This was replicated in a targeted study of 162 severe and 247 mild IAV patients. This genetic association in humans is highly biologically plausible: TMPRS22 has been shown to play a role in haemagluttinin cleavage, an important step in IAV replication. Additionally, mice lacking this gene are strongly protected from IAV infection [74–76].

#### SP-B

This genome-wide array also identified a SNP in pulmonary-surfactant-associated protein B (SP-B), rs1130866, as a potential association. This SNP was genotyped in a targeted study of 111 severe and 185 mild IAV patients to replicate the finding [77]. Again, this is a plausible association with severe disease: SP-B forms a key part of pulmonary surfactant and is essential for lung function. A subset of the same protein family, SP-A and SP-D, have been shown to initiate and enhance immune cell ingestion and killing (opsonisation) of pathogens and play a role in the progression of IAV in mice [78]. A polymorphism associated with SP-B, rs1130866 [77], has also been associated with COPD in several cohorts [79].

#### GLDC

Susceptibility to severe H1N1 infection was analysed in a recent genome-wide study (integrated with data on genetic variants associated with altered gene expression) which implicated an intronic SNP of GLDC, rs1755609-G [80]. The GLDC gene encodes glycine decarboxylase, also known as the P protein of the glycine cleavage system, a pathway in glycine metabolism [81]. The association was replicated by targeted genotyping in a larger cohort of 174 patients suffering severe IAV infection and 258 mildly infected controls. The risk variant corresponds to higher GLDC expression in lymphoblastoid cell lines and human lung tissues. Consistent with this effect, inhibition of GLDC in cultured bronchial epithelium using siRNA or a specific inhibitor, aminooxyacetic acid (AOAA), leads to an increased type I IFN response and a restriction of viral replication in vitro. This effect on viral restriction was seen with both H1N1 and H7N9, and the allele genotype was replicated in susceptibility cohorts for both viruses. The protective effect of AOAA against H1N1 was shown in mice to be comparable with that of zanamivir.

#### Galectin-1

Susceptibility to severe H7N9 was examined in a GWAS performed with 102 patients and 106 controls who worked with poultry. This study identified rs13057866, associated with Galectin-1 (LGALS1), as a potential susceptibility factor. LGALS1 is a lectin that may have a role in modulating cell-cell and cell-matrix interactions. The study further demonstrated that genetic variants of LGALS1, including rs4820294 and rs13057866, lead to higher expression of LGALS1 protein in human cells, possibly leading to a protective effect. Carriers of the rs4820294/rs2899292 GG haplotype were found to have higher LGALS1 protein in lymphoblastoid cells and expression levels of LGALS1 in human lung correlated with the rs4820294 SNP [82].

## Conclusions

The role of host factors in susceptibility suggests a clinically important conclusion: there is something unusual about the small minority of patients who develop critical illness following IAV. Therefore, extrapolating from human challenge and primary care studies of viral clearance is very likely to lead to error. Viral clearance among critically ill patients is slow and incomplete [83]. Hence, the critically ill population should be regarded—by definition—as highly abnormal hosts.

Susceptible hosts may have impaired intracellular controls of viral replication (e.g. IFITM3, TMPRS22 variants), defective interferon responses (e.g. GLDC, IRF7/9 variants), or defects in cell-mediated immunity with increased baseline levels of systemic inflammation (obesity, pregnancy, advanced age). In the context of any of these susceptibility mechanisms, failure to clear the virus is an expected consequence, indicating that a full course of effective antiviral therapy is likely to benefit this population. In the future, understanding the biological mechanisms of susceptibility to severe IAV may yield therapeutic targets to modify the biology of the susceptible hosts in critical care and render them resilient.

## Data Availability

Not applicable.
